# Body Composition During Pregnancy: Longitudinal Changes and Method Comparisons

**DOI:** 10.1007/s43032-020-00141-6

**Published:** 2020-01-28

**Authors:** Marja Bosaeus, Ulrika Andersson-Hall, Louise Andersson, Therese Karlsson, Lars Ellegård, Agneta Holmäng

**Affiliations:** 1 Department of Physiology, Institute of Neuroscience and Physiology, Sahlgrenska Academy, University of Gothenburg, Box 432, SE-405 30 Gothenburg, Sweden; 2 Department of Internal Medicine and Clinical Nutrition, Institute of Medicine, Sahlgrenska Academy, University of Gothenburg, Gothenburg, Sweden; 3 Department of Endocrinology, Diabetes and Metabolism, Sahlgrenska University Hospital, Gothenburg, Sweden

**Keywords:** Body composition, Pregnancy, Air displacement plethysmography, Quantitative magnetic resonance, Bioelectrical impedance analysis

## Abstract

The Pregnancy Obesity Nutrition and Child Health study is a longitudinal study of reproductive health. Here we analyzed body composition of normal-weight and obese Swedish women by three methods during each trimester of pregnancy. Cross-sectional and longitudinal fat mass estimates using quantitative magnetic resonance (QMR) and bioelectrical impedance analysis (BIA) (Tanita MC-180MA-III) were compared with fat mass determined by air displacement plethysmography (ADP) in pregnancy weeks 8–12, 24–26, and 35–37 in normal-weight women (*n* = 122, BMI = 22.1 ± 1.6 kg/m^2^) and obese women (*n* = 29, BMI = 34.6 ± 3.6 kg/m^2^). ADP results were calculated from pregnancy-adjusted fat-free mass densities. Mean fat mass by QMR and ADP were similar in obese women, although with wide limits of agreement. In normal-weight women, QMR overestimated mean fat mass in all trimesters, with systematic overestimation at low fat mass values in trimesters 1 and 3. In obese women, fat mass by BIA was grossly underestimated and imprecise in all trimesters, especially at higher values in trimester 2. In normal-weight women, fat mass by BIA was moderately lower than by ADP in trimester 1, similar in trimester 2, and moderately higher in trimester 3. QMR and ADP assessed fat mass changes similarly in obese women, whereas BIA overestimated fat mass changes in normal-weight women. Mean fat mass and fat mass changes by QMR and pregnancy-adjusted ADP were similar in pregnant obese women. Mean fat mass by QMR and fat mass changes by BIA were higher than corresponding values determined by pregnancy-adjusted ADP in normal-weight women.

## Background

Appropriate gestational weight gain (GWG)—reflecting contributions from fetus, placenta, mammary glands, uterus, fat tissue, amniotic fluid, extracellular fluids, and blood volume expansion—is important for a successful pregnancy and avoiding complications such as pre-eclampsia and low birth weight [1]. GWG varies considerably but is generally lower at higher BMIs [1].

The gold standard for measuring body composition during pregnancy is a four-component model based on body weight (BW), densitometry, total body water (TBW) determined by stable isotope dilution, and bone mineral content [2] measured before [3] or after pregnancy [3, 4]. However, isotope dilution is time consuming and requires a laboratory. The three-component model, using densitometry and TBW, controls for interindividual variation in fat-free mass (FFM) hydration. Body composition technology such as dual-energy X-ray absorptiometry, yields values for fat mass (FM) and bone mineral. Although the increased risks to both mother and fetus are small, methods involving ionizing radiation should be avoided. The three-component model controls for biological variability in TBW and thus should be more valid than the densitometry two-component model; however, additional control for interindividual variability in bone mineral content—a feature of the four-component model—is considered to achieve little extra accuracy [5]. There is therefore a need for safe and easy methods to measure body composition during pregnancy.

Densitometry, by underwater weighing or air displacement plethysmography (ADP), is based on the relationship between assumed constant densities of FM and FFM and measures both maternal and fetal tissues [6]. The increased hydration and change in density of FFM during pregnancy [6] is a true challenge for calculation of two-component models. Theoretical models of a reference body have been used to estimate FFM densities at various stages of pregnancy [7, 8]. Others have used three-component [9, 10] or four-component [11, 12] models to assess sequential changes in FFM hydration during pregnancy and published hydration constants or FFM densities. New densitometry equations based on estimated changes in FFM hydration and density during pregnancy [7] have been validated for use in late pregnancy [11] and for assessing FM changes during pregnancy [12]. ADP is a user-friendly densitometry method and safe during pregnancy. Although few validation studies of ADP during pregnancy have been performed, it implements the same densitometry theories as hydrodensitometry/underwater weighing to assess a two-component body composition model and a comparative study of two and three-compartment models deemed ADP with adjustments for FFM hydration as the best model for use in late pregnancy [13].

Bioelectrical impedance analysis (BIA) is quick, safe during pregnancy, and user-friendly. However, neither BIA nor ADP can separate maternal from fetal tissues [6]. Although BIA has advantages, the abnormal fluid distribution during pregnancy makes different impedance methods either inappropriate or in need of further validation [14]. Whole-body impedance is mainly predicted by impedance in the limbs [15]; however, a large amount of water is located in the trunk during pregnancy [16]. Thus, segmental impedance measurement might be advantageous [17], particularly in late pregnancy. Cross-sectional TBW has been validated in early and late pregnancy [16, 18]. Also, descriptive longitudinal impedance pregnancy data has been published [19, 20] but no previous study evaluated both cross-sectional and longitudinal FM method comparisons by BIA in normal weight and obese women during pregnancy.

Quantitative magnetic resonance (QMR) uses nuclear magnetic resonance (NMR) [21] to measure FM, lean mass, free water, and TBW [22] of the maternal–fetal unit. QMR has not been used or validated during pregnancy.

Since quicker and more user friendly methods for determining body composition during pregnancy need to be evaluated, our aims in this study were (1) to compare body composition by BIA and QMR to pregnancy-adjusted ADP and (2) to compare FM changes during pregnancy. If consistent accuracy was found for BIA, this would facilitate quick, easy, and inexpensive body composition measurements in reproductive health care.

## Methods

### Study Design and Participants

Between April 2009 and April 2014, pregnant with normal weight and obese pregnant women were recruited for the Pregnancy Obesity Nutrition and Child Health (PONCH) study at Sahlgrenska University Hospital, Gothenburg, Sweden [23, 24]. PONCH is a longitudinal randomized dietary intervention study of normal weight (BMI 18.5–24.9 kg/m^2^) and obese (BMI > 30 kg/m^2^) Swedish pregnant women. The PONCH study was approved by the ethics committee at the University of Gothenburg, number 402-08. Participants received oral and written information and signed an informed consent document before entering the study.

The inclusion criteria were age 20–45 years and self-reported BMI of 18.5–24.9 kg/m^2^ or ≥ 30 kg/m^2^ at the time of recruitment. Self-reported BMI was only used for inclusion. Exclusion criteria were self-reported diabetes, use of neuroleptic drugs, non-European descent, and vegetarianism or veganism. Pregnancy in trimester 1 was dated from the first day of the last menstruation. Pregnancy in trimesters 2 and 3 was dated by ultrasound in the general maternal health care.

**Fig. 1:**
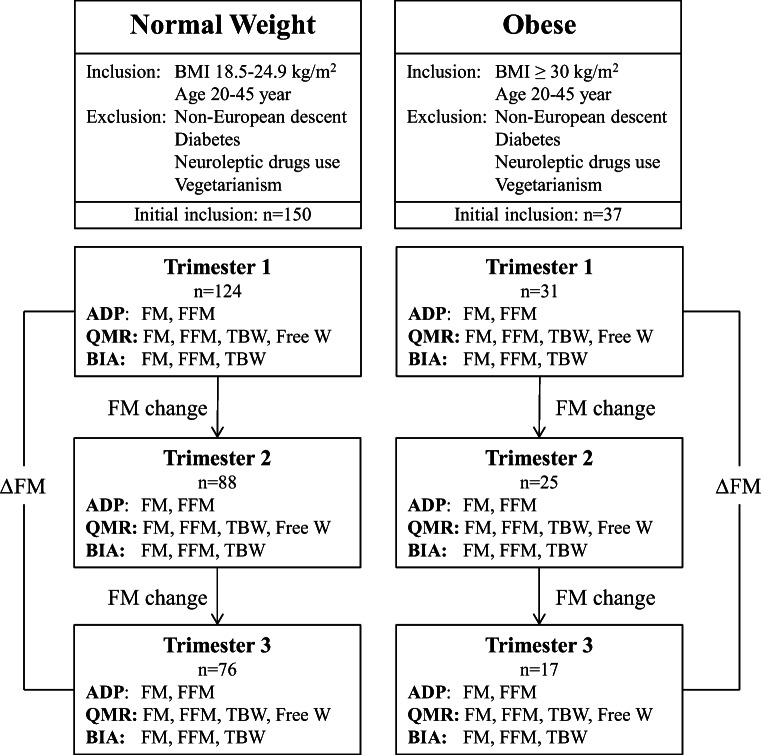
Study flow chart for the Pregnancy Obesity Nutrition and Child Health study*.* FM, fat mass. ADP, air displacement plethysmography. QMR, quantitative magnetic resonance. BIA, bioelectrical impedance analysis. Δ FM was calculated as the FM difference in trimesters 1 and 3. Participants (*n*) denotes number of women at each trimester study visit

Women in trimester 1 of pregnancy were recruited through written information at maternity care centers in Gothenburg, postings on public billboards, and advertisement on a website for pregnant women. Participants were randomized to a control group or an intervention group. Both the control and intervention groups attended three study visits during pregnancy. The first study visit took place during trimester 1 (weeks 8–12) (Fig. 1). Follow-up study visits were done in trimesters 2 (weeks 24–26) and 3 (weeks 35–37). Women were asked to fast overnight before study visits. The study visits took place in the morning at the Sahlgrenska University Hospital and included body composition measurements by ADP, QMR, and BIA; all measurements were completed within 2 h. At each visit, blood samples were collected, and the women filled in questionnaires. The visits were the same for both groups, but with the addition of dietary counseling by registered dieticians in the intervention group as previously described [23, 24]. There were no differences in any of the outcomes of the present study between intervention and control groups within each BMI category, and the data has therefore been pooled (fat mass was for normal weight women 17.3 ± 3.9 kg in control vs 16.9 ± 5.0 kg in intervention groups in trimester 1 and 20.3 ± 4.3 kg vs 21.3 ± 5.6 kg in trimester 3, *p* > 0.38; fat mass was for obese women 44.3 ± 8.5 kg in control vs 46.0 ± 11.4 kg in intervention groups in trimester 1 and 47.6 ± 11.5 kg vs 49.6 ± 7.0 kg in trimester 3, *p* > 0.67) The numbers of normal weight and obese participants were 124 and 31, respectively, during trimester 1; 88 and 25 during trimester 2; and 76 and 17 during trimester 3 (Fig. 1). However, because of missing data in different body composition measures, the number of women analyzed was lower in different analyses. The age of included women was 31.2 ± 3.6 and 31.6 ± 3.3 years in normal weight and obese groups, respectively. BMI was 22.1 ± 1.6 and 34.6 ± 3.6 kg/m^2^ in normal weight and obese groups, respectively.

ADP with equations adjusted for FFM densities according to van Raaij et al. (ADP_vR_) [7] was used as our reference method. The reason for using ADP_vR_ as reference method was that pregnancy-adjusted densitometry has been validated for use in late pregnancy [11] and for assessing FM changes during pregnancy [12], and provided the best estimate of maternal FM compared with post-pregnancy DXA values in an evaluation of two- and three-compartment models [13]. These studies also showed that mean FM in trimester 3 and FM gain during pregnancy were higher in the Siri densitometry two-component model than in the four-component model, whereas the densitometry two-component model with van Raaij correction was accurate [11, 12].

### ADP

Body composition was measured by ADP with the Bod Pod Gold Standard system (Bod Pod 2007 A, Life Measurement, Concord, CA) and software versions 4.2.1 and 5.2.0. The coefficient of variation from duplicate measurements on our equipment in 22 individuals was 2.4%. Software quality checks and scale calibrations were done routinely. Subjects were weighed dressed in bathing cap and underwear. Height was measured to the nearest centimeter. The Bod Pod system measured BW with a modified Tanita BWB-627-A electronic scale. The BodPod system performed two measurements of body volume and if the two measurements were deemed inconsistent by the software (criteria for inconsistency not specified by the manufacturer), a third measurement was done. Predicted lung gas volume was used. FM density was kept constant at 0.90 kg/L.

In trimester 1, the FM equation for pregnancy week 10 published by van Raaij et al. [7] was used (FM_ADPvR1_). BW_1_ is body weight (kg) and Db_1_ is body density (kg/L) in trimester 1:

$$ {\mathrm{FM}}_{\mathrm{ADPvR}1}\ \left(\mathrm{kg}\right)={\mathrm{BW}}_1/100\times \left(496.4/{\mathrm{Db}}_1-451.6\right) $$

$$ {\mathrm{FFM}}_{\mathrm{ADPvR}1}\ \left(\mathrm{kg}\right)={\mathrm{BW}}_1-{\mathrm{FM}}_{\mathrm{ADPvR}1} $$

In trimester 2, FFM density in pregnancy week 25 was 1.095 kg/L, estimated from Fig. 1 of van Raaij et al. [7]. Based on D_FFM_ 1.095 kg/L and D_FM_ 0.90 kg/L, the following FM_ADPvR2_ equation was developed, where BW_2_ is body weight (kg) and Db_2_ is body density (kg/L) in trimester 2:

$$ {\mathrm{FM}}_{\mathrm{ADPvR}2}\ \left(\mathrm{kg}\right)={\mathrm{BW}}_2\times \left(5.0538/{\mathrm{Db}}_2-4.6154\right) $$

$$ {\mathrm{FFM}}_{\mathrm{ADPvR}2}\ \left(\mathrm{kg}\right)={\mathrm{BW}}_2-{\mathrm{FM}}_{\mathrm{ADPvR}2} $$

In trimester 3, FM was calculated according to the equation published by Hopkinson et al. [11] (FM_ADPvR3_), who estimated D_FFM_ from van Raaij et al. in pregnancy week 36 at 1.089 kg/L. BW_3_ is body weight (kg) and Db_3_ is body density (kg/L) in trimester 3:

$$ {\mathrm{FM}}_{\mathrm{ADPvR}3}\ \left(\mathrm{kg}\right)={\mathrm{BW}}_3\times \left(5.19/{\mathrm{Db}}_3-4.76\right) $$

$$ {\mathrm{FFM}}_{\mathrm{ADPvR}3}\ \left(\mathrm{kg}\right)={\mathrm{BW}}_3-{\mathrm{FM}}_{\mathrm{ADPvR}3} $$

### QMR

To measure body composition by QMR, we used an EchoMRI-AH instrument (EchoMRI, Houston, TX) and a homogeneous low-intensity magnetic field of 0.0065 T. The measurements were done in a box (inside dimensions 198 × 61 × 61 cm). Subjects were measured in underwear/light clothing and weighed with a Tanita BWB-620 scale to the nearest 0.05 kg (maximum weight 200 kg). The subject was placed in a comfortable position halfway between sitting and lying and moved into the measurement box. The door of copper net was closed to prevent external electric interference. The integration time for one measurement was set to 3 min. The measurement voxel was the entire volume inside the box. The system was tested daily using 10 gal of canola oil at room temperature (22 ± 1 °C). To further decrease the influence of measurement noise, each examination consisted of four contiguous measurements. Final body composition was calculated as the mean of the last three measurements, unless noted otherwise. One normal weight woman each in trimesters 1 and 2 had only one QMR measurement, which was used for analysis. One normal weight woman each in trimesters 1 and 2 had only two QMR measurements, and data from the second measurement were used. The coefficient of variation from triplicate measurements was 0.3% [25].

Fat, free water, and muscle mass have different NMR signals [21] and linear regression analysis formulas calibrated against canola oil, tap water, and lean animal tissues are used to calculate fat, free water, and lean mass [26]. TBW is derived from the difference between the total amount of protons and the fat found by regression analysis and thus includes both free water and water in lean mass [26]. The output from a measurement was total fat mass (FM_QMR_), total lean tissue mass, total body water mass (TBW_QMR_), and free water mass (FreeW_QMR_). However, lean tissue mass by QMR is not equivalent to non-FM [21]. Therefore, to allow comparison to FFM by ADP, FFM by QMR was calculated as follows, using BW measured with a Tanita BWB-620 scale (BW_QMR_):

$$ {\mathrm{FFM}}_{\mathrm{QMR}}\ \left(\mathrm{kg}\right)={\mathrm{BW}}_{\mathrm{QMR}}-{\mathrm{FM}}_{\mathrm{QMR}} $$

In a few cases, BW_QMR_ by Tanita BWB-620 was missing. Therefore, BW by Tanita MC-180MA III (BIA) was used to calculate FFM_QMR_ in two normal weight women in trimester 1 and one normal weight woman each in trimesters 2 and 3. Also, FFM_QMR_ was calculated from the first QMR FM measurement in one normal weight woman in trimester 1, and BW by Tanita MC-180MA III (BIA) and the first QMR FM measurement were used to calculate FFM_QMR_ in one normal weight woman in trimester 2.

### BIA

For BIA measurements, we used a Tanita MC-180MA III multi-frequency, eight-electrode segmental body composition analyzer, which has a reported accuracy of 2% [27]. The coefficient of variation from duplicate measurements on our equipment in 22 individuals was 3.0%. Subjects were measured in underwear, standing barefoot on toe and heel electrodes, and holding the handgrips with arms hanging down a few centimeters from the hip. The eight-electrode method enables segmental impedance measurement. Since ADP reference data do not include segmental analysis, segmental body composition results by BIA are not presented here. MC-180MA measurements were obtained at 5, 50, 250, and 500 kHz at a current of 90 μA or less. Minimum weight graduation was 0.05 kg. Body composition by BIA was calculated from the measured impedance by the manufacturer’s proprietary software [27]. Height to the nearest centimeter, BW, age, and segmental impedance values were used to calculate FM_BIA_, FFM_BIA_, and TBW_BIA_ [27]. The operator manual provided no further information on body composition calculations.

### Statistical Analyses

IBM SPSS Statistics versions 21, 22, and 23 were used for statistical analyses. Descriptive data are presented as mean ± SD. Data from the maximum number of women were used to analyze baseline age, BMI, GWG, Δ FM, and Δ FFM. GWG, Δ FM by ADP and Δ FFM by ADP were calculated as trimester 3 minus trimester 1. GWG was calculated using BW from ADP measurements. FM changes between trimesters 1 and 2 and between trimesters 2 and 3 were calculated as the latest trimester minus the previous one. Pearson’s correlation was used to examine the relationship between GWG and both Δ FM (ADP_vR_) and Δ FFM (ADP_vR_). Comparisons of FM determined by different methods in each trimester were analyzed by paired-samples *t* test. FM differences between methods were also analyzed with boxplots and with Bland-Altman plots; in both analyses, ADP_vR_ was the reference method. Bland-Altman plots were analyzed by linear regression to determine whether mean FM by ADP_vR_ + QMR or by ADP_vR_ + BIA could explain FM bias by QMR or by BIA. Limits of agreement in Bland-Altman plots were calculated as mean ± 2 SD. The ability of the methods to measure FM changes between trimesters was analyzed using paired-samples *t* test; ADP_vR_ was used as reference method. *P* < 0.05 was considered significant.

## Results

### Body Composition During Pregnancy

**Fig. 2:**
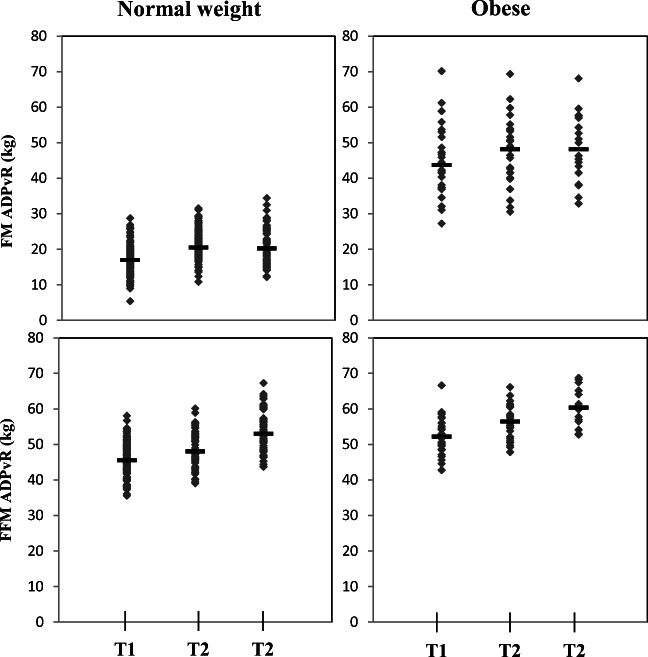
Fat mass and fat-free mass in normal weight and obese women during pregnancy. Individually plotted fat mass and fat-free mass as measured by air displacement plethysmography for normal weight and obese pregnant women from the PONCH study cohort. Black lines represent median. FM, fat mass; FFM, fat free mass; ADP_vR_, air displacement plethysmography calculated with fat-free mass densities adjusted according to van Raaij et al. [7]; T1, trimester 1; T2, trimester 2; T3, trimester 3

Body composition measured by ADP, QMR, and BIA is presented in Table 1, and FM and FFM by ADP_vR_ are visualized in Fig. 2. For normal weight women, GWG was 11.5 ± 2.9 kg, Δ FM 4.1 ± 3.0 kg, and Δ FFM 7.4 ± 2.2 kg. For obese women, GWG was 8.8 ± 4.9 kg, Δ FM 2.2 ± 4.1 kg, and Δ FFM 6.6 ± 3.5 kg. GWG correlated with Δ FM in normal weight women (*r* = 0.727, *p* = 0.000; *n* = 71, Pearson’s correlation) and obese women (*r* = 0.714, *p* = 0.001; *n* = 17). GWG also correlated with Δ FFM in normal weight women (*r* = 0.338, *p* = 0.004; *n* = 71) and obese women (*r* = 0.555, *p* = 0.021; *n* = 17).

**Table 1:** Body composition during pregnancy in normal weight and obese women from the PONCH study

	Normal weight women						Obese women					
	Trimester						Trimester					
	1		2		3		1		2		3	
	Mean	SD	Mean	SD	Mean	SD	Mean	SD	Mean	SD	Mean	SD
ADP_vR_	*n* = 64						*n* = 17					
FM_ADPvR_ (kg)	16.9	4.3	20.4	4.3	21.0	5.0	45.7	9.0	47.3	9.0	47.9	9.6
FFM_ADPvR_ (kg)	46.2	4.8	48.9	4.7	53.6	5.2	53.4	4.4	55.9	4.3	60.0	4.6
QMR	*n* = 50						*n* = 14					
FM_QMR_ (kg)	17.3	3.7	21.3	3.9	23.0	4.4	47.8	8.0	49.3	7.7	50.7	7.9
FFM_QMR_(kg)	45.7	3.8	48.2	3.7	51.6	4.0	53.3	5.2	56.0	5.3	59.5	5.4
TBW_QMR_ (kg)	30.7	2.7	32.5	2.4	35.1	2.8	33.7	3.5	36.3	3.8	38.7	3.8
FreeW_QMR_ (kg)	0.5	0.1	1.2	0.3	1.3	0.3	0.5	0.1	1.2	0.2	1.5	0.4
BIA	*n* = 42						*n* = 10					
FM_BIA_ (kg)	16.1	3.9	20.3	3.8	23.3	4.4	42.8	7.1	45.6	7.1	45.0	10.6
FFM_BIA_ (kg)	47.5	4.5	49.6	4.3	52.0	4.3	60.6	7.5	61.6	6.4	67.1	8.6
TBW_BIA_ (kg)	34.2	3.4	35.6	3.0	37.3	3.1	43.5	5.4	44.2	4.5	48.7	7.2

*ADP*_*vR*_*,* air displacement plethysmography calculated with the FFM densities suggested by van Raaij et al. [7]. *FM*, fat mass; *FFM*, fat-free mass; *QMR*, quantitative magnetic resonance; *TBW*, total body water; *FreeW*, free water; *BIA*, bioelectrical impedance analysis

### Comparison of Body Composition Methods During Pregnancy

**Fig. 3:**
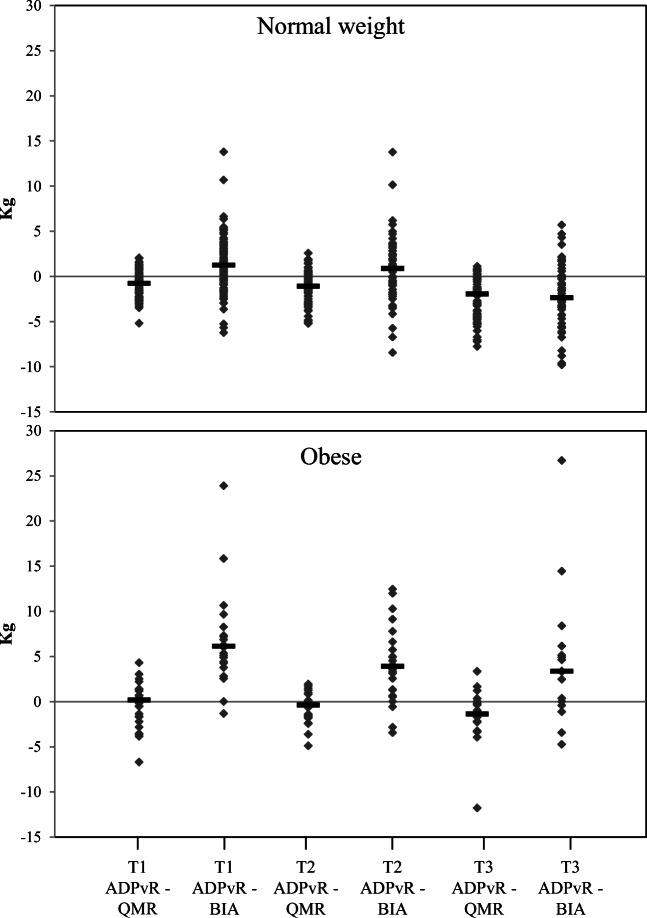
Differences between body composition methods in normal weight and obese women during pregnancy. The differences are plotted individually for each women and the black line represents the median. FM, fat mass; ADP_vR_, air displacement plethysmography calculated with fat-free mass densities adjusted according to van Raaij et al. [7]; QMR, quantitative magnetic resonance; BIA, bioelectrical impedance analysis; T1, trimester 1; T2, trimester 2; T3, trimester 3

**Fig. 4:**
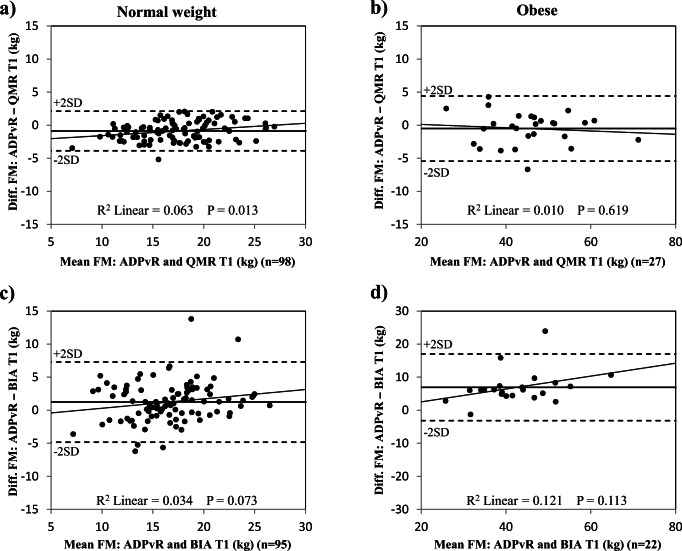
Comparison of body composition methods in normal weight and obese women in trimester 1. Bland-Altman plots illustrating the differences in fat mass (FM) measured by air displacement plethysmography adjusted according to fat-free mass densities reported from Raaij et al. [7] (ADP_vR_) and by quantitative magnetic resonance (QMR) in normal weight women (**a**) and obese women (**b**) and bioelectrical impedance analysis (BIA) in normal weight women (**c**) and obese women (**d**). Black horizontal line represents mean difference. T1, trimester 1. Note that the vertical scale is − 30 to +30 kg in Fig. 4d

**Fig. 5:**
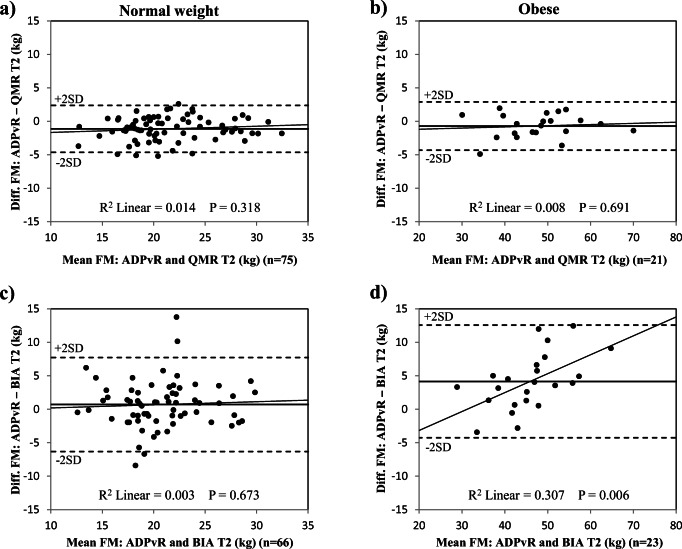
Comparison of body composition methods in normal weight and obese women in trimester 2. Bland-Altman plots illustrating the differences in fat mass (FM) measured by air displacement plethysmography adjusted according to fat-free mass densities from van Raaij et al. [7] (ADP_vR_) and by quantitative magnetic resonance (QMR) in normal weight women (**a**) and obese women (**b**) and by bioelectrical impedance analysis (BIA) in normal weight women (**c**) and obese women (**d**). Black horizontal line represents mean difference. T2, trimester 2

**Fig. 6:**
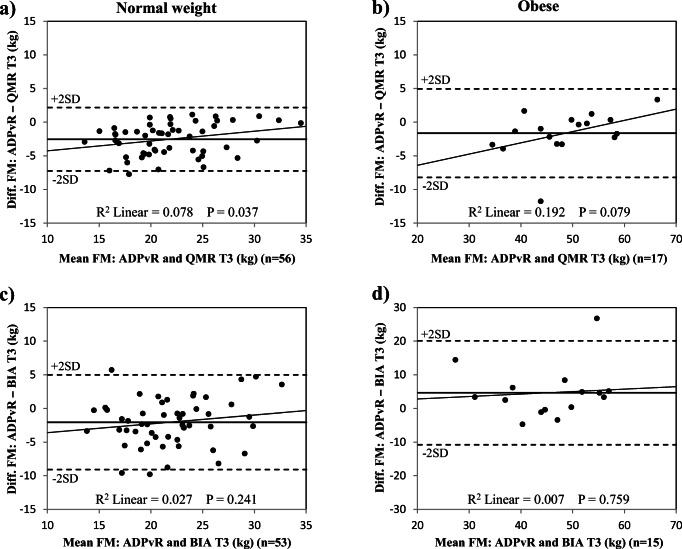
Comparison of body composition methods in normal weight and obese women in trimester 3. Bland-Altman plots illustrating the differences in fat mass (FM) measured by air displacement plethysmography adjusted according to fat-free mass densities from van Raaij et al. [7] (ADP_vR_) and by quantitative magnetic resonance (QMR) in normal weight women (**a**) and obese women (**b**) and by bioelectrical impedance analysis (BIA) in normal weight women (**c**) and obese women (**d**). Black horizontal line represents mean difference. T3, trimester 3. Note that the vertical scale is − 30 to + 30 kg in Fig. 6d

FM assessment methods are compared in Table 2. Differences between methods are illustrated in Fig. 3. Bland-Altman plots for method comparisons are shown in Figs. 4, 5, and 6. All differences presented in this section are statistically significant unless stated otherwise.

**Table 2:** Comparison of body composition methods during pregnancy in normal weight and obese women

Fat mass difference	Normal weight women				Obese women			
	Mean	SD	*p*	*n*	Mean	SD	*p*	*n*
Trimester 1								
ADP_vR_−QMR (kg)	− 0.9	1.5	0.000	98	− 0.5	2.5	0.287	27
ADP_vR_−BIA (kg)	1.2	3.0	0.000	95	6.9	5.0	0.000	22
Trimester 2								
ADP_vR_−QMR (kg)	− 1.1	1.7	0.000	75	− 0.7	1.8	0.091	21
ADP_vR_−BIA (kg)	0.7	3.5	0.112	66	4.2	4.2	0.000	23
Trimester 3								
ADP_vR_−QMR (kg)	− 2.5	2.4	0.000	56	− 1.6	3.3	0.057	17
ADP_vR_−BIA (kg)	− 2.1	3.5	0.000	53	4.7	7.7	0.035	15

*ADP*_*vR*_, air displacement plethysmography calculated with the fat-free mass densities suggested by van Raaij et al. [7]. *QMR*, quantitative magnetic resonance; *BIA*, bioelectrical impedance analysis. Statistical significance was determined by paired-samples *t* test

In trimester 1, mean FM was slightly higher by QMR and moderately lower by BIA than by ADP_vR_ in normal weight women. In obese women, mean FM by QMR was not different from ADP_vR_, but with wide limits of agreement (up to 9.9 kg) (Fig. 4b), whereas mean FM was much lower by BIA than by ADP_vR_ (Table 2).

In trimester 2, mean FM by QMR was moderately higher than by ADP_vR_ in normal weight women. Mean FM was not different from FM by ADP_vR_ in obese women but with wide limits of agreement (up to 7.2 kg) (Fig. 5b). Mean FM was much lower by BIA than by ADP_vR_ in obese women. Mean FM by BIA did not differ from FM by ADP_vR_ in normal weight women, again with wide limits of agreement (up to 14 kg) (Fig. 5c).

In trimester 3, mean FM by QMR or by BIA was moderately higher than ADP_vR_ in normal weight women (Table 3). In obese women, mean FM was much lower by BIA than by ADP_vR_; however, FM by QMR and by ADP_vR_ did not differ (Table 3) but had wide limits of agreement (up to 13.2 kg) (Fig. 6b).

**Table 3:** Comparison of body composition methods for assessment of fat mass changes during pregnancy

FM change difference	Normal weight women				Obese women			
	Mean	SD	*p*	*n*	Mean	SD	*p*	*n*
Trimester 1–2								
ADP_vR_−QMR (kg)	− 0.4	1.6	0.069	68	− 0.3	2.5	0.571	18
ADP_vR_−BIA (kg)	− 0.7	2.6	0.040	61	− 2.9	3.6	0.003	18
Trimester 2–3								
ADP_vR_−QMR (kg)	− 1.3	1.9	0.000	48	− 0.04	1.7	0.934	15
ADP_vR_−BIA (kg)	− 2.5	3.2	0.000	43	0.8	7.1	0.662	14
Trimester 1–3								
ADP_vR_−QMR (kg)	− 1.6	2.0	0.000	49	− 0.5	2.9	0.495	15
ADP_vR_−BIA (kg)	− 3.0	3.2	0.000	49	− 1.3	7.6	0.630	9

*FM*, fat mass; *ADP*_*vR*_, air displacement plethysmography calculated with the fat-free mass densities suggested by van Raaij et al. [7]. *QMR*, quantitative magnetic resonance; *BIA*, bioelectrical impedance analysis. Statistical significance was determined by paired samples *t* test

Bland-Altman plots for FM by QMR in trimester 1 showed that QMR slightly overestimated FM at low mean FM values in normal weight women (R^2^ = 0.063, *p* = 0.013, *n* = 98) (Fig. 4a). BIA increased FM underestimation at higher mean values in obese women in trimester 2 (R^2^ = 0.307, *p* = 0.006, *n* = 23) (Fig. 5d). Bland-Altman plots for FM by QMR in trimester 3 showed that QMR slightly overestimated FM at low mean FM values in normal weight women (R^2^ = 0.078, *p* = 0.037, *n* = 56); a similar trend was detected in obese women (R^2^ = 0.192, *p* = 0.079, *n* = 17) (Fig. 6a, b).

### Comparison of Body Composition Methods for Assessing FM Changes During Pregnancy

Methods of assessing changes in FM are compared in Table 3. All differences in this section are statistically significant unless stated otherwise.

In normal weight women, FM change estimated by BIA was slightly overestimated compared with FM change by ADP_vR_ between trimesters 1 and 2; moderately overestimated between trimesters 2 and 3; and greatly overestimated between trimesters 1 and 3 (Table 3). Compared with ADP_vR_, FM change estimated by QMR was not different between trimester 1 and trimester 2, but was moderately higher between trimesters 2 and 3 and between trimesters 1 and 3.

In obese women, change in FM estimated by both QMR and by BIA between trimesters 1 and 2, trimesters 2 and 3, and trimesters 1 and 3 were not significantly different from FM change by ADP_vR_; however, between trimesters 1 and 2, FM changes were much higher by BIA than by ADP_vR_.

## Discussion

In this study, we analyzed body composition by ADP, QMR, and BIA in each trimester of pregnancy in normal weight and obese women. We found that mean FM by QMR was similar to FM calculated from pregnancy-adjusted ADP in each trimester in obese women, although with wide limits of agreement, but not in normal weight women. Furthermore, mean FM by BIA was similar to FM calculated from pregnancy-adjusted ADP in trimester 2 in normal weight women, with wide limits of agreement, but not in any other trimester in normal weight or obese women. FM changes assessed by QMR and by pregnancy-adjusted ADP were similar throughout pregnancy in obese women, but only between trimesters 1 and 2 in normal weight women. Mean FM changes during pregnancy were higher by BIA than by pregnancy-adjusted ADP in normal weight women. Mean FM changes by BIA and by pregnancy-adjusted ADP were similar between trimesters 1 and 3 and between trimesters 2 and 3 in obese women; however, the BIA values varied considerably. Thus, the Tanita MC-180MA III bioimpedance device with current software is unsuitable for assessing body composition during pregnancy.

In populations with women with a mean normal BMI TBW gain by BIA was 7–8 L [19, 20], FM gain by dilution was 3–4 kg [28, 29], and FM gain by a three-component model was 6 kg [30]. FM gain by four-component models in women classified by BMI was 4–5 kg and TBW gain 7 L in normal weight women [3, 4], whereas obese women also gained 7 L of TBW, but only 0.2 kg of FM [4]. The FM gain in normal weight women was in agreement with the present study, but obese women in our study gained slightly more fat.

In our study, BIA mainly failed to similarly assess cross-sectional body composition, when FM was evaluated against pregnancy-adjusted ADP. On the other hand, FM changes by BIA and pregnancy-adjusted ADP were similar in late pregnancy, although only in obese women and with large variations, whereas early pregnancy changes were overestimated. When bioimpedance studies are compared, an important source of error could be the use of different equipment, and especially built-in software and algorithms inaccessible to users. The eight-electrode BIA system we evaluated has only been used once before during pregnancy. In that study, body composition changes were estimated between pregnancy weeks 28 and 37 and the bioimpedance devices differed. Although the measurement occasions and BIA analysis were not fully comparable to those in the present study, the increase in maternal FM was similar to that in obese women in our study but lower in normal weight women [31].

Cross-sectional TBW by bioimpedance devices (Xitron 4000 and 4200) during pregnancy has been evaluated in earlier studies [16, 18], and descriptive impedance pregnancy data has also been published [19, 20]. The results from these studies are quite similar to ours in early pregnancy, but quite large differences are found in late pregnancy in all studies. In a methodological study that estimated TBW by BIA and compared the results with estimates obtained with reference methods (isotope and bromide dilution) during pregnancy [16], average TBW by BIA was in agreement with reference values in gestational week 14 but was significantly lower than those in week 32. The study concluded that the BIA technique could estimate TBW changes accurately in early pregnancy but not the TBW gain during the whole pregnancy [16].

In our study, FM changes assessed by QMR were similar to pregnancy-adjusted ADP throughout pregnancy in obese women, but only between trimesters 1 and 2 in normal weight women. QMR overestimated mean FM in normal weight women in all trimesters. TBW gain estimated by QMR was approximately 4 kg in normal weight women and 5 kg in obese women. TBW by QMR is estimated from the total amount of protons and the fat found [26] and thus could avoid problems related to changes in FFM hydration during pregnancy. Although biased in normal weight and obese nonpregnant women [25], cross-sectional QMR may be suitable for use during pregnancy, as FM results are independent from changes in FFM hydration [22] and body shape should not affect NMR [32]. However, FM measures by QMR can be slightly affected by free water [22] and thus might derange TBW measures during pregnancy. QMR has not previously been applied or proved to be useful during pregnancy. Our results suggest that QMR is potentially useful, easy, comfortable, and fast for studies during pregnancy; however, further work is needed before it can be generally used in such studies.

We chose to use theoretical FFM density values, which could be a source of error, especially in obesity, which already in the nonpregnant state is characterized by increased extracellular water (ECW) [33] and therefore lowered FFM density [34]. However, theoretical FFM densities have been widely used [11, 12, 35, 36], and the healthy normal weight population used here should probably hardly differ from the Dutch women in the van Raaij studies. Furthermore, the biological variability of hydration constants could reduce the accuracy of two-component models in early pregnancy [10]. Also, van Raaij et al. discussed possible interindividual variation in composition of the FFM gain and whether the ratio of pre-pregnancy FFM to FFM gain would affect FM calculations, but concluded that the FFM densities based on a reference woman should be appropriate in most cases [7].

### Limitations and Strengths

There are other possible limitations and sources of error, besides FFM hydration. First, the two-component densitometry model, BIA, and QMR cannot separate fetal tissues from maternal tissues. Second, we used predicted thoracic gas volume. However, use of predicted rather than actual thoracic gas volume has little effect on calculation of body fat percent during pregnancy [36] and thus is probably acceptable. Third, while the normal weight group was sufficiently powered (post-hoc power analysis achieved a power of 1.0 for both BIA-ADPvr and QMR-ADPvr in trimester 3), the obese group was underpowered (power of 0.6 for BIA-ADPvr and 0.5 for QMR-ADPvr in trimester 3). However, in obese women, the mean difference in FM by QMR and by pregnancy-adjusted ADP was only 0.5–0.7 kg in trimesters 1 and 2, a small nonsignificant difference. Finally, this body composition analysis was part of a larger study with many objectives, methods, and tests for the women included. Therefore, the accuracy of body composition methods was not evaluated against the time-consuming gold standard method which would be of great interest for future studies that focus solely on body composition. The prospective randomized design of the larger study might also has affected the fat mass increase in the women, although no differences in outcome was seen between women in the intervention arm compared with the control arm.

This study had three strengths. First, measurements were made in the last part of each trimester, allowing longitudinal follow-up. Second, the women were classified into BMI groups allowing separate analysis within normal weight and obese categories. Finally, this is the first study to publish QMR body composition measurements during pregnancy.

## Conclusions

In this study, we present body composition data determined in all trimesters of pregnancy by ADP, QMR, and BIA in normal weight and obese women. Compared with previous longitudinal studies, fat accumulation during pregnancy was close to expected values in normal weight women, but slightly more than expected in obese women. QMR, a new precise method for assessing body fat, produced results similar to FM by ADP according to van Raaij et al. [7], although with wide limits of agreement, and without systematic bias in obese women. Thus, with the wide QMR limits of agreement for mean FM in obese women (although much narrower than by Tanita MC-180MA III), individual assessment seems uncertain when compared with FM by pregnancy-adjusted ADP. Mean FM by QMR was overestimated throughout pregnancy in normal weight women, and the small bias was systematic in early and late pregnancy. QMR should be further validated before clinical use, preferably against a four-component model. Bioimpedance by Tanita MC-180MA III with in-built software produced imprecise measurements of mean FM, which differed from mean FM by pregnancy-adjusted ADP. Thus, BIA with the Tanita MC-180MA III should not be used for cross-sectional assessment of FM measures during pregnancy. Mean FM changes by QMR and pregnancy-adjusted ADP were similar throughout pregnancy in obese women but only in early pregnancy in normal weight women. Longitudinal FM changes during pregnancy should not be assessed by Tanita MC-180MA III in normal weight or obese women.

## Abbreviations

ADP: air displacement plethysmography
BIA: bioelectrical impedance analysis
BW: body weight
D: density
FFM: fat-free mass
FM: fat mass
FreeW: free water
GWG: gestational weight gain
NMR: nuclear magnetic resonance
PONCH: Pregnancy Obesity Nutrition and Child Health study
QMR: quantitative magnetic resonance
TBW: total body water
T1: trimester 1
T2: trimester 2
T3: trimester 3
vR: van Raaij

## References

[1] Cedergren M (2006). Effects of gestational weight gain and body mass index on obstetric outcome in Sweden. Int J Gynaecol Obstet.

[2] Lederman SA. Pregnancy. In: Heymsfield S, Lohman T, Wang Z, Going SB, editors. Human body composition, vol. 918: Human kinetics; 2005. p. 299–312.

[3] Butte NF, Ellis KJ, Wong WW, Hopkinson JM, Smith EB (2003). Composition of gestational weight gain impacts maternal fat retention and infant birth weight. Am J Obstet Gynecol.

[4] Lederman SA, Paxton A, Heymsfield SB, Wang J, Thornton J, Pierson RN (1997). Body fat and water changes during pregnancy in women with different body weight and weight gain. Obstet Gynecol.

[5] Withers RT, LaForgia J, Pillans R, Shipp NJ, Chatterton BE, Schultz CG, Leaney F (1998). Comparisons of two-, three-, and four-compartment models of body composition analysis in men and women. J Appl Physiol.

[6] Widen E, Gallagher D (2014). Body composition changes in pregnancy: measurement, predictors and outcomes. Eur J Clin Nutr.

[7] Van Raaij J, Peek M, Vermaat-Miedema SH, Schonk CM, Hautvast J (1988). New equations for estimating body fat mass in pregnancy from body density or total body water. Am J Clin Nutr.

[8] Fidanza F. The density of fat-free body mass during pregnancy. Int J Vitam Nutr Res. 1987;57(1):104–4. 3583588

[9] Catalano PM, Wong WW, Drago NM, Amini SB (1995). Estimating body composition in late gestation: a new hydration constant for body density and total body water. Am J Physiol Endocrinol Metab.

[10] Lof M, Forsum E (2004). Hydration of fat-free mass in healthy women with special reference to the effect of pregnancy. Am J Clin Nutr.

[11] Hopkinson JM, Butte NF, Ellis KJ, Wong WW, Puyau MR, Smith E (1997). Body fat estimation in late pregnancy and early postpartum: comparison of two-, three-, and four-component models. Am J Clin Nutr.

[12] Kopp-Hoolihan L, Van Loan M, Wong W, King J (1999). Fat mass deposition during pregnancy using a four-component model. J Appl Physiol.

[13] Marshall NE, Murphy EJ, King JC, Haas EK, Lim JY, Wiedrick J, Thornburg KL, Purnell JQ (2016). Comparison of multiple methods to measure maternal fat mass in late gestation. Am J Clin Nutr.

[14] Kyle UG, Bosaeus I, De Lorenzo AD (2004). Bioelectrical impedance analysis—part II: utilization in clinical practice. Clin Nutr.

[15] De Lorenzo A, Andreoli A (2003). Segmental bioelectrical impedance analysis. Curr Opin Clin Nutr Metab Care.

[16] Lof M, Forsum E (2004). Evaluation of bioimpedance spectroscopy for measurements of body water distribution in healthy women before, during, and after pregnancy. J Appl Physiol.

[17] Fattah C, Farah N, Barry S, O'connor N, Stuart B, Turner M (2009). The measurement of maternal adiposity. J Obstet Gynaecol.

[18] Van Loan M, Kopp L, King J, Wong W, Mayclin P (1995). Fluid changes during pregnancy: use of bioimpedance spectroscopy. J Appl Physiol.

[19] Larciprete G, Valensise H, Vasapollo B (2003). Body composition during normal pregnancy: reference ranges. Acta Diabetol.

[20] Valensise H, Andreoli A, Lello S, Magnani F, Romanini C, De Lorenzo A (2000). Multifrequency bioelectrical impedance analysis in women with a normal and hypertensive pregnancy. Am J Clin Nutr.

[21] Taicher GZ, Tinsley FC, Reiderman A, Heiman ML (2003). Quantitative magnetic resonance (QMR) method for bone and whole-body-composition analysis. Anal Bioanal Chem.

[22] Bosy-Westphal A, Müller MJ (2015). Assessment of fat and lean mass by quantitative magnetic resonance: a future technology of body composition research?. Curr Opin Clin Nutr Metab Care..

[23] Karlsson T, Andersson L, Hussain A, Bosaeus M, Jansson N, Osmancevic A, Hulthén L, Holmäng A, Larsson I (2015). Lower vitamin D status in obese compared with normal-weight women despite higher vitamin D intake in early pregnancy. Clin Nutr.

[24] Bosaeus M, Hussain A, Karlsson T (2015). A randomized longitudinal dietary intervention study during pregnancy: effects on fish intake, phospholipids, and body composition. Nutr J.

[25] Bosaeus M, Karlsson T, Holmang A, Ellegard L (2014). Accuracy of quantitative magnetic resonance and eight-electrode bioelectrical impedance analysis in normal weight and obese women. Clin Nutr.

[26] Gallagher D, Thornton JC, He Q, Wang J, Yu W, Bradstreet TE, Burke J, Heymsfield SB, Rivas VM, Kaufman R (2010). Quantitative magnetic resonance fat measurements in humans correlate with established methods but are biased. Obesity..

[27] Tanita. Multi-frequency body composition analyzer MC-180MA III Instruction Manual. In. Tokyo, Japan.

[28] Eriksson B, Löf M, Olausson H, Forsum E (2010). Body fat, insulin resistance, energy expenditure and serum concentrations of leptin, adiponectin and resistin before, during and after pregnancy in healthy Swedish women. Br J Nutr.

[29] Goldberg GR, Prentice AM, Coward WA, Davies HL, Murgatroyd PR, Wensing C, Black AE, Harding M, Sawyer M (1993). Longitudinal assessment of energy expenditure in pregnancy by the doubly labeled water method. Am J Clin Nutr.

[30] Forsum E, Sadurskis A, Wager J (1988). Resting metabolic rate and body composition of healthy Swedish women during pregnancy. Am J Clin Nutr.

[31] Farah N, Stuart B, Donnelly V, Kennelly MM, Turner MJ (2011). The influence of maternal body composition on birth weight. Eur J Obstet Gynecol Reprod Biol.

[32] Galgani JE, Smith SR, Ravussin E (2011). Assessment of EchoMRI-AH versus dual-energy X-ray absorptiometry to measure human body composition. Int J Obes.

[33] Waki M, Kral JG, Mazariegos M, Wang J, Pierson R, Heymsfield S (1991). Relative expansion of extracellular fluid in obese vs. nonobese women. Am J Physiol Endocrinol Metab.

[34] Le Carvennec M, Fagour C, Adenis-Lamarre E, Perlemoine C, Gin H, Rigalleau V (2007). Body composition of obese subjects by air displacement plethysmography: the influence of hydration. Obesity..

[35] Forsum E, Henriksson P, Löf M (2014). The two-component model for calculating total body fat from body density: an evaluation in healthy women before, during and after pregnancy. Nutrients..

[36] Henriksson P, Löf M, Forsum E (2013). Assessment and prediction of thoracic gas volume in pregnant women: an evaluation in relation to body composition assessment using air displacement plethysmography. Br J Nutr.

